# Cadmium Disrupts the Balance between Hydrogen Peroxide and Superoxide Radical by Regulating Endogenous Hydrogen Sulfide in the Root Tip of *Brassica rapa*

**DOI:** 10.3389/fpls.2017.00232

**Published:** 2017-02-21

**Authors:** Wenjing Lv, Lifei Yang, Cunfa Xu, Zhiqi Shi, Jinsong Shao, Ming Xian, Jian Chen

**Affiliations:** ^1^Institute of Food Quality and Safety, Jiangsu Academy of Agricultural SciencesNanjing, China; ^2^College of Horticulture, Nanjing Agricultural UniversityNanjing, China; ^3^Key Lab of Food Quality and Safety of Jiangsu Province – State Key Laboratory Breeding Base, Jiangsu Provincial Department of Agriculture and ForestryNanjing, China; ^4^Central Laboratory, Jiangsu Academy of Agricultural ScienceNanjing, China; ^5^Agricultural Products Quality and Safety Superivision, Inspection, and Testing Center, Ministry of AgricultureNanjing, China; ^6^Department of Chemistry, Washington State University, PullmanWA, USA

**Keywords:** cadmium, hydrogen sulfide, hydrogen peroxide, superoxide radical, root tip, *Brassica rapa*

## Abstract

Cd (cadmium) stress always alters the homeostasis of ROS (reactive oxygen species) including H_2_O_2_ (hydrogen sulfide) and $O_{2}^{•–}$ (superoxide radical), leading to the oxidative injury and growth inhibition in plants. In addition to triggering oxidative injury, ROS has been suggested as important regulators modulating root elongation. However, whether and how Cd stress induces the inhibition of root elongation by differentially regulating endogenous H_2_O_2_ and $O_{2}^{•–}$, rather than by inducing oxidative injury, remains elusive. To address these gaps, histochemical, physiological, and biochemical approaches were applied to investigate the mechanism for Cd to fine-tune the balance between H_2_O_2_ and $O_{2}^{•–}$ in the root tip of *Brassica rapa*. Treatment with Cd at 4 and 16 μM significantly inhibited root elongation, while only 16 μM but not 4 μM of Cd induced oxidative injury and cell death in root tip. Fluorescent and pharmaceutical tests suggested that H_2_O_2_ and $O_{2}^{•–}$ played negative and positive roles, respectively, in the regulation of root elongation in the presence of Cd (4 μM) or not. Treatment with Cd at 4 μM led to the increase in H_2_O_2_ and the decrease in $O_{2}^{•–}$ in root tip, which may be attributed to the up-regulation of *Br_UPB1s* and the down-regulation of their predicted targets (four peroxidase genes). Cd at 4 μM resulted in the increase in endogenous H_2_S in root tip by inducing the up-regulation of *LCDs* and *DCDs*. Treatment with H_2_S biosynthesis inhibitor or H_2_S scavenger significantly blocked Cd (4 μM)-induced increase in endogenous H_2_S level, coinciding with the recovery of root elongation, the altered balance between H_2_O_2_ and $O_{2}^{•–}$, and the expression of *Br_UPB1s* and two peroxidase genes. Taken together, it can be proposed that endogenous H_2_S mediated the phytotoxicity of Cd at low concentration by regulating *Br_UPB1s*-modulated balance between H_2_O_2_ and $O_{2}^{•–}$ in root tip. Such findings shed new light on the regulatory role of endogenous H_2_S in plant adaptions to Cd stress.

## Introduction

Reactive oxygen species (ROS), a set of active forms of molecular oxygen (O_2_) occurred in plant cells, comprise both free radical (e.g., $O_{2}^{•–}$, superoxide radical; OH, hydroxyl radical) and non-radical forms (e.g., H_2_O_2_, hydrogen peroxide; ^1^O_2_, singlet oxygen) (Gill and Tuteja, 2010). ROS accumulation can be frequently induced by environmental stimuli, which further results in oxidative injury in plants. However, ROS can act as second messengers in the regulation of plant intrinsic physiology and development under both stress and normal environmental conditions (Apel and Hirt, 2004). For instance, ROS has been suggested as one of the key workers for the regulation of plant root development. In the primary root of *Arabidopsis*, $O_{2}^{•–}$ located in the elongation zone (EZ) positively regulates root elongation, while H_2_O_2_ located in the differentiation zone (DZ) negatively regulates root elongation (Dunand et al., 2007). Additionally, both H_2_O_2_ and $O_{2}^{•–}$ are indispensable for the emergence of lateral root in *Arabidopsis* (Manzano et al., 2014). ROS functions as core modulator of sophisticated network of signaling pathways in plants, but the regulation of the exact nature of ROS-mediated signaling network remains largely obscured (Bhattacharjee, 2012). It has been evidenced that a basic helix-loop-helix transcription factor UPBEAT1 (UPB1) is an important regulator of ROS signaling during root development. UPB1 can directly suppress the expression of several peroxidases (*Per39, Per40*, and *Per57*) that modulate the balance between H_2_O_2_ and $O_{2}^{•–}$ (Tsukagoshi et al., 2010). The alteration of ROS balance resulted from the stimulation of UPB1 activity accelerates the onset of cell differentiation, leading to the inhibition of root elongation (Tsukagoshi et al., 2010). The reduced lateral root number was also found in both *UPB1*-overexpressing plant and *per57* mutant, suggesting that UPB1-mediated ROS signaling is also important to control lateral root growth (Manzano et al., 2014). Nevertheless, UPB1/peroxidase-mediated ROS signaling acts independently of auxin signaling that is a typical regulator of root development (Tsukagoshi et al., 2010; Manzano et al., 2014).

Cadmium (Cd) contamination has been drawing great attention worldwide because large amounts of Cd have been released into the ecosystem due to both natural and anthropogenic activities (Satarug et al., 2010). Cd-induced phytotoxicity has been closely linked to the over-generation of ROS, leading to oxidative injury, lipid peroxidation, cell death, and growth stunt (DalCorso et al., 2010; Lin and Aarts, 2012; Andresen and Küpper, 2013). In general, excessive Cd at toxic dosage induces remarkable increases in both H_2_O_2_ and $O_{2}^{•–}$ in plants (Xu et al., 2012; Pérez-Chaca et al., 2014). $O_{2}^{•–}$ induced by Cd is mainly originated from NADPH oxidase (Jakubowska et al., 2015), while H_2_O_2_ is produced by the univalent reduction of $O_{2}^{•–}$ (Gill and Tuteja, 2010). In Cd-treated plants, ROS-mediated oxidative stress can be regulated by several factors, such as nitric oxide (NO) (Rodríguez-Serrano et al., 2009; Pérez-Chaca et al., 2014), Ca^2+^ (Rodríguez-Serrano et al., 2009), an oxidative stress-related Abc1-like protein (AtOAS1) (Jasinski et al., 2008), etc. In some cases, H_2_O_2_ and $O_{2}^{•–}$ can be differentially regulated by Cd stress. For instance, Cd induces two waves of ROS in the roots of *Glycine max*, which the maximum accumulation of H_2_O_2_ appears faster than that of $O_{2}^{•–}$ (Pérez-Chaca et al., 2014). In the roots of *G. max* and *Cucumis sativus*, Cd stimulates H_2_O_2_ production whereas it inhibits $O_{2}^{•–}$ production (Heyno et al., 2008). However, whether and how ROS act as signaling molecule rather than a trigger of oxidative stress to regulate root growth under Cd exposure remains obscured.

Hydrogen sulfide (H_2_S) acting as an important signaling molecule in mammals has been highly appreciated for its clinical relevance (Wang, 2010; Kimura, 2011; Kimura et al., 2012; Vandiver and Snyder, 2012). The emerging role of H_2_S in the modulation of various plant physiological pathways has been revealing, which is involved in the regulation of stomatal closure, phototosynthesis, seed germination, flower senescence, root development, and responses to abiotic stress, etc (García-Mata and Lamattina, 2013; Lisjak et al., 2013; Fotopoulos et al., 2015; Jia et al., 2015). H_2_S can be produced by _L_-cysteine desulfhydrase (LCD, EC4.4.1.1) and D-cysteine desulfhydrase (DCD, EC4.4.1.15) in plants (Papenbrock et al., 2007). Large amounts of reports suggest that exogenous application of H_2_S can protect plants from metal toxicity by inhibiting the over-generation of H_2_O_2_ or $O_{2}^{•–}$ (Zhang et al., 2008, 2010a,b; Chen et al., 2013; Bharwana et al., 2014; Shi et al., 2014). In our previous study, the endogenous H_2_S detected selectively by a specific fluorescent probe Washington Stat Probe 1 (WSP-1) is essential for root growth under selenium stress by scavenging the over-generated total ROS and $O_{2}^{•–}$ (Chen et al., 2014). H_2_S has been suggested to promote root organogenesis while H_2_O_2_ and $O_{2}^{•–}$ play vital role in the regulation of root growth (Zhang et al., 2009; Tsukagoshi et al., 2010). The antioxidant roles of H_2_S in scavenging ROS have been highlighted in both plants and mammals (Ju et al., 2013; Hancock and Whiteman, 2014). Nevertheless, whether and how endogenous H_2_S differentially fine-tunes the balance between H_2_O_2_ and $O_{2}^{•–}$ *in vivo* remains unclear.

In this work, we investigated the possible link between H_2_S and ROS signaling in the regulation of root elongation under Cd exposure. First, we found a disturbance of the balance between H_2_O_2_ and $O_{2}^{•–}$ without any oxidative injury in root treated with Cd at a specific concentration. The involvement of the endogenous H_2_S in the regulation of the above process was further elucidated. To get deeper insights into the link between of H_2_S and ROS signaling, the expression of *UPB1* and its possible targets were studied under the application of H_2_S-synthesizing inhibitor or H_2_S scavenger in root in the presence of Cd. Finally, the possible mechanisms driving these physiological processes, and their significance, were discussed.

## Materials and Methods

### Plant Culture, Treatment, and Chemicals

Seeds of *B. rapa* (LvLing) seeds were surface-sterilized with 1% NaClO for 10 min followed by washing with distilled water. Seeds were germinated for 1 day in the dark on the floating plastic nets. Then the selected identical seedlings with radicles 0.5 cm were transferred to another Petri dish containing various treatment solutions in a chamber with a photosynthetic active radiation of 200 μmol/m^2^/s, a photoperiod of 12 h, and the temperature at 25 ± 1°C.

Seedling roots were exposed to CdCl_2_ (cadmium chloride) with different concentrations (0–32 μM) for various treatment time (0–72 h). PAG (DL-propargylglycine) (0.05–0.2 mM) and HT (hypotaurine) (0.1–0.4 mM) were used as H_2_S biosynthesis inhibitor and H_2_S scavenger, respectively (Chen et al., 2014). DPI (diphenylene iodonium) and KI (potassium iodide) were used as NADPH oxidase inhibitor and H_2_O_2_ scavenger, respectively (Tsukagoshi et al., 2010). The treatment solution is composed of different chemicals mentioned above alone or their combinations according to the experimental design. After treatments, the roots were washed with distilled water for physiological, histochemical, and biochemical analysis.

### Histochemical Analysis

The intracellular H_2_S was visualized using specific fluorescent probe WSP-1 [3′-methoxy-3-oxo-3H-spiro[isobenzofuran-1,9′-xanthen]-6′-yl 2-(pyridin-2-yldisulfanyl) benzoate] *in situ* according to our previous method (Li et al., 2014). The roots of seedlings after treatments were incubated at 20 mM Hepes-NaOH (pH 7.5) buffer solution containing 20 μM of WSP-1 at 25°C for 40 min. Then the roots were washed with distilled water three times and were visualized immediately by a fluorescence microscope with a 465/515 nm and an excitation/emission filter set (ECLIPSE, TE2000-S, Nikon). The relative fluorescent density of the fluorescent images was analyzed using Image-Pro Plus 6.0 (Media Cybernetics, Inc.).

Intracellular $O_{2}^{•–}$ was visualized *in situ* using specific fluorescent probe DHE (dihydroethidium) *in situ* described by Yamamoto et al. (2002). The roots of seedlings after treatment were incubated in 15 μM of DHE at 25°C for 15 min. Then the roots were rinsed with distilled water for three times and were visualized (excitation 535 nm and emission 610 nm) by a fluorescence microscope (ECLIPSE, TE2000-S, Nikon). The relative fluorescent density of the fluorescent images was analyzed using Image-Pro Plus 6.0 (Media Cybernetics, Inc.).

Intracellular H_2_O_2_ was visualized *in situ* using specific fluorescent probe HPF (3′-(p-hydroxyphenyl) fluorescein) *in situ* described by Dunand and Crevecoeur (Dunand et al., 2007). The roots of seedlings after treatment were incubated in 5 μM of HPF at 25°C for 15 min. Then the roots were rinsed with distilled water for three times and were visualized (excitation 490 nm and emission 515 nm) by a fluorescence microscope (ECLIPSE, TE2000-S, Nikon). The relative fluorescent density of the fluorescent images was analyzed using Image-Pro Plus 6.0 (Media Cybernetics, Inc.).

Histochemical detection of lipid peroxidation was achieved by using Schiff′s regent as described by Wang and Yang (2005). The roots of seedlings after treatment were incubated in Schiff′s regent for 20 min. Then the stained roots were rinsed with a solution containing 0.5% (w/v) K_2_S_2_O_5_ (prepared in 0.05 M of HCl) until the root color became light red. After that, the roots were imaged by using a stereoscopic microscope (SteREO Discovery.V8, ZEISS).

Histochemical detection of loss of plasma membrane integrity was performed by using Evans blue as described by Yamamoto et al. (2001). The roots of seedlings after treatment were incubated in Evans blue solution (0.025%, w/v) for 20 min. After that, the roots were rinsed with distilled water for three times followed by imaging with a stereoscopic microscope (SteREO Discovery.V8, ZEISS).

Histochemical detection of cell death was performed by using Trypan blue (Duan et al., 2010). The roots of seedlings after treatment were incubated in Trypan 10 mg/mL of blue solution for 20 min. After that, the roots were rinsed with distilled water for three times followed by imaging with a stereoscopic microscope (SteREO Discovery.V8, ZEISS).

### Analysis of Transcripts

Total RNA was extracted from root tip using Trizol (Invitrogen) according to the manufacturer’s instructions. The possible genomic DNA was removed from extracted RNA samples by using Recombinant DNase I (RNase-free) (TaKaRa Bio Inc, China). Reverse transcription was performed at 42°C in 25 μl reaction mixture including 3 μg of RNA, 0.5 μg of oligo (dT) primers, 12.5 nmol of dNTPs, 20 units of RANase inhibitor and 200 units of M-MLV. The first cDNA was used as a template for polymerase chain amplification and to analyze the transcripts of genes by using real-time quantitative reverse transcription-polymerase chain reaction (qRT-PCR) (Applied Biosystems 7500 Fast Real-Time PCR System, LifeTechnologies^TM^). with SYBR Premix Ex Taq^TM^ (TaKaRa Bio Inc, China) according to the manufacturer’s instructions. The qPCR procedure was as follows: initial denaturation at 95°C for 30 s, followed by 40 cycles of 95°C for 5 s, 60°C for 30 s, and 72°C for 30 s. Data were collected and analyzed by using ABI 7500 software (v. 2.0.6, Applied Biosystems) based on 2^-ΔΔ^*^C^*^T^ threshold cycle method (Livak and Schmittgen, 2001). The relative abundance of *Actin* was determined and used as the internal standard to normalize the data. The expression levels of corresponding genes are presented as values relative to the control samples under the indicated conditions. The primers designed for the amplification of the genes are listed in Supplementary Table 1.

### Cluster analysis

Hierarchical cluster analysis for different parameters was performed by using Cluster 3.0^1^. The generated tree figures were displayed by using Java Treeview^2^ (de Hoon et al., 2004; Shi et al., 2014).

### Statistical analysis

Each result was presented as the mean ± standard deviation (SD) of at least three replicated measurement. The significant differences between treatments were statistically evaluated by SD and one-way analysis of variance (ANOVA) using SPSS 2.0. The data between two specific different treatments were compared statistically by ANOVA, followed by *F*-test if the ANOVA result is significant at *P* < 0.05. For multiple comparison analysis, least significant difference test (LSD) was performed on all data following ANOVA tests to test for significant (*P* < 0.05) differences among different treatments.

## Results

### Cd at Specific Concentration Inhibited Root Growth Without Inducing Oxidative Injury and Cell Death

**FIGURE 1:**
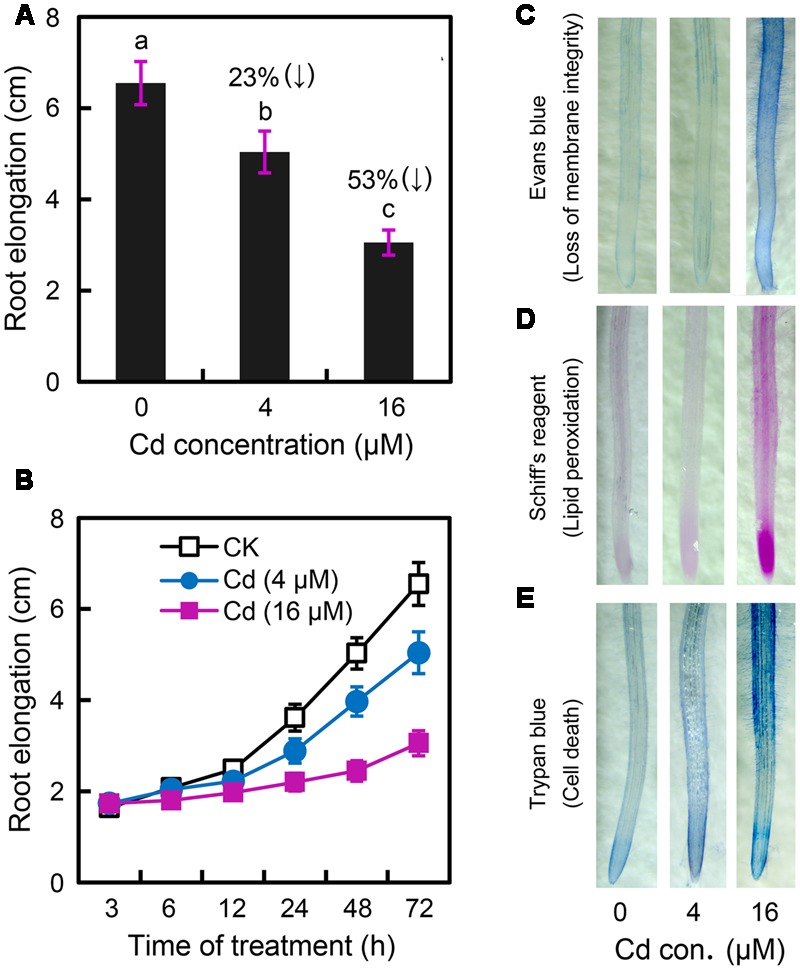
**Effect of Cadmium (Cd) stress on the root elongation and physiological changes in the root tip of *Brassica rapa*. (A)** The root elongation was obtained when the roots of seedlings were exposed to 0, 4, and 16 μM of CdCl_2_ (cadmium chloride) for 72 h. The numbers in the top of columns indicate the inhibitory percentage of the treatments as compared to the control. The mean values of five replicates followed by different letters indicate significance of difference between the treatments [*P* < 0.05, analysis of variance (ANOVA), least significant difference test (LSD)]. **(B)** The roots of seedlings were exposed to 0, 4, and 16 μM of CdCl_2_. The average root elongation was obtained from five replicates at 3, 6, 12, 24, 48, and 72 h, respectively. **(C–E)** The root elongation was obtained when the roots of seedlings were exposed to 0 (control), 4, and 16 μM of CdCl_2_ for 72 h. Then the roots were histochemically stained with Evans blue **(C)**, Shiff’s reagent **(D)**, and trypan blue **(E)**, respectively, for imaging.

In order to determine the effect of Cd exposure on root elongation, the roots of *B. rapa* were exposed to CdCl_2_ (2–32 μM) for 72 h. CdCl_2_ at 4–32 μM significantly inhibited root growth in a dose-dependent manner (Supplementary Figure 1A). Root elongation significantly decreased by 23 and 53% at 4 and 16 μM Cd levels, respectively, as compared to the control (**Figure 1A**). Cd stress always induces oxidative injury, leading to cell death in plants (Andresen and Küpper, 2013). Membrane lipid peroxidation, indicated by MDA (malondiadehyde) content, is the typical consequence of Cd-induced oxidative injury. Cd at high concentrations (8–32 μM), but not low concentrations (2–4 μM), resulted in remarkable increase in MDA content in root as compared to control (Supplementary Figure 1B). Thus, Cd at 4 and 16 μM were considered to induce slight and relatively severe stress in root, respectively. In a time-course experiment, exposure of Cd at 16 μM for only 6 h began to significantly inhibit root elongation, while root elongation treated with Cd at 4 μM began to decrease remarkably after 24 h (**Figure 1B**). The peroxidation of membrane lipids and the loss of plasma membrane integrity were tested *in vivo* using histochemical staining with Shiff’s reagent and Evans blue, respectively. Root tips treated with 4 μM of Cd and the control group had only slight staining. Nevertheless, root tips treated with Cd at 16 μM were stained extensively (**Figures 1C,D**). Trypan blue was applied to indicate cell death in root under Cd exposure. Root tip treated with Cd at 16 μM showed extensive blue staining as compared to the slight staining of control group and 4 μM of Cd treatment (**Figure 1E**). These results suggested that Cd at 4 μM impeded root elongation without inducing oxidative damage and cell death in the root of *B. rapa*.

### Cd Disturbed ROS Balance in Root Tip

**FIGURE 2:**
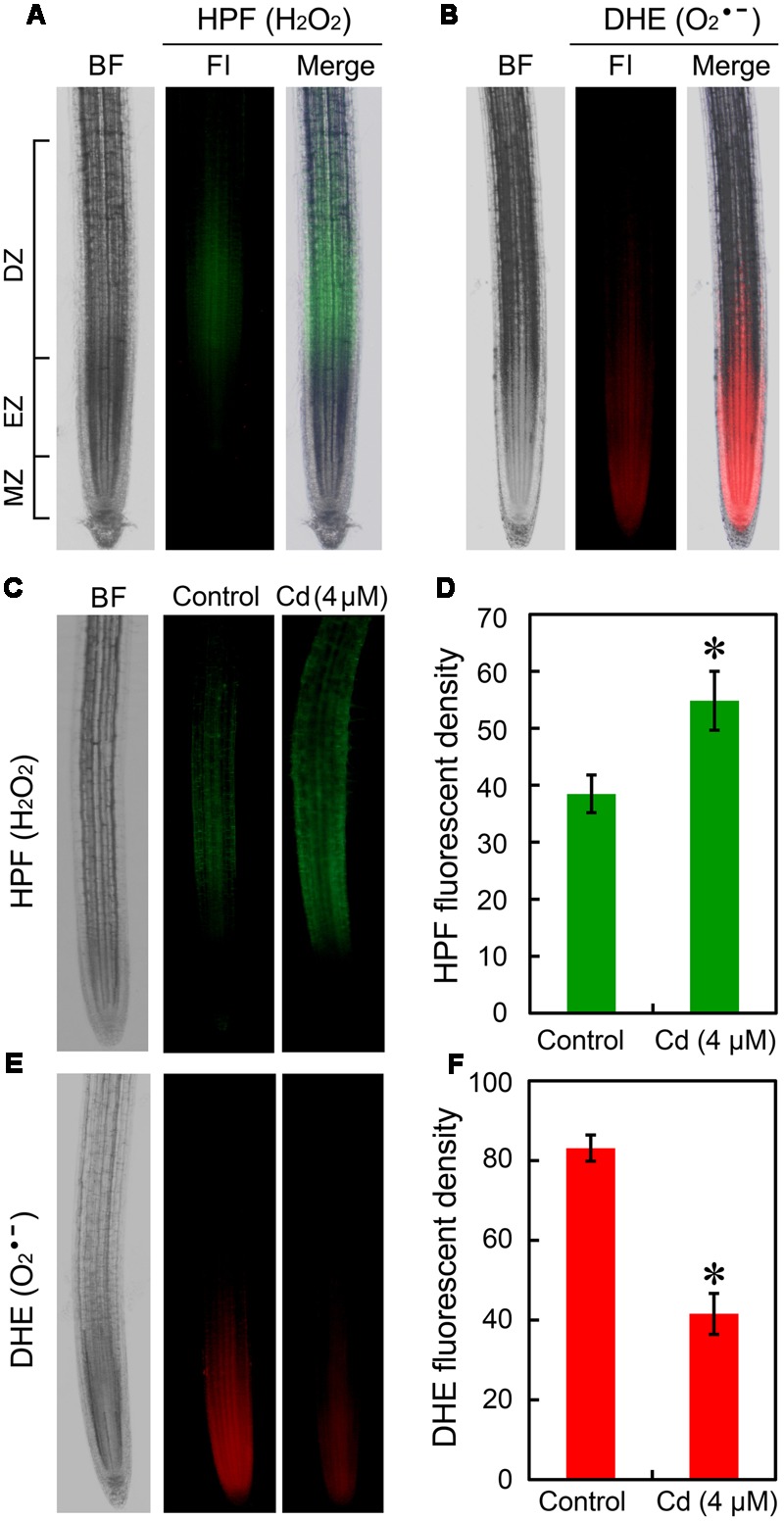
**Effect of Cd (4 μM) on the content of endogenous H_2_O_2_ and $O_{2}^{•–}$ in the root tip of *B. rapa*. (A)** The roots of seedlings were loaded with HPF (3′-(p-hydroxyphenyl) fluorescein) for fluorescent imaging of endogenous H_2_O_2_ in root tip. **(B)** The roots of seedlings were loaded with DHE (dihydroethidium) for fluorescent imaging of endogenous $O_{2}^{•–}$ in root tip. **(C)** After treated with 0 (control) and 4 μM of CdCl_2_ for 72 h, the roots were loaded HPF for fluorescent imaging of endogenous H_2_O_2_ in root tip. **(D)** The HPF fluorescent density was calculated corresponding to the images obtained from **(C)**. **(E)** After treated with 0 (control) and 4 μM of CdCl_2_ for 72 h, the roots were loaded with DHE for fluorescent imaging of endogenous H_2_O_2_ in root tip. **(F)** The DHE fluorescent density was calculated corresponding to the images obtained from **(E)**. *Asterisk* indicates that mean values of three replicates are significantly different between treatments and control (*P* < 0.05) in **(D,F).**

The location of H_2_O_2_ and $O_{2}^{•–}$ in root tip were fluorescently detected *in vivo* by using HPF and DHE, respectively. In normal growth conditions, H_2_O_2_ indicated as green fluorescence mainly distributed in DZ while $O_{2}^{•–}$ indicated as red fluorescence was located in EZ and meristem zone (MZ) (**Figures 2A,B**). Compared to the control group, treatment with Cd at 4 μM resulted in significant increase in H_2_O_2_ and remarkable decrease in $O_{2}^{•–}$ in root tip (**Figures 2C–F**). To confirm the above results, H_2_O_2_ and $O_{2}^{•–}$ were also stained with DAB and NBT, respectively. We obtained similar results for the location and Cd-induced changes of H_2_O_2_ and $O_{2}^{•–}$ as compared to the fluorescently detective methods (Supplementary Figures 2A,B). Then we tested the effect of Cd at 16 μM on ROS balance. The results from histochemical analysis indicated that Cd at 16 μM triggered considerable accumulation of both H_2_O_2_ and $O_{2}^{•–}$ in root tips (Supplementary Figures 2A,B), which may evidence the oxidative injury and cell death in root tip treated with 16 μM of Cd. The measurement of the content of H_2_O_2_ and $O_{2}^{•–}$ in root tip also showed similar results with histochemical analysis (Supplementary Figures 2C,D).

**FIGURE 3:**
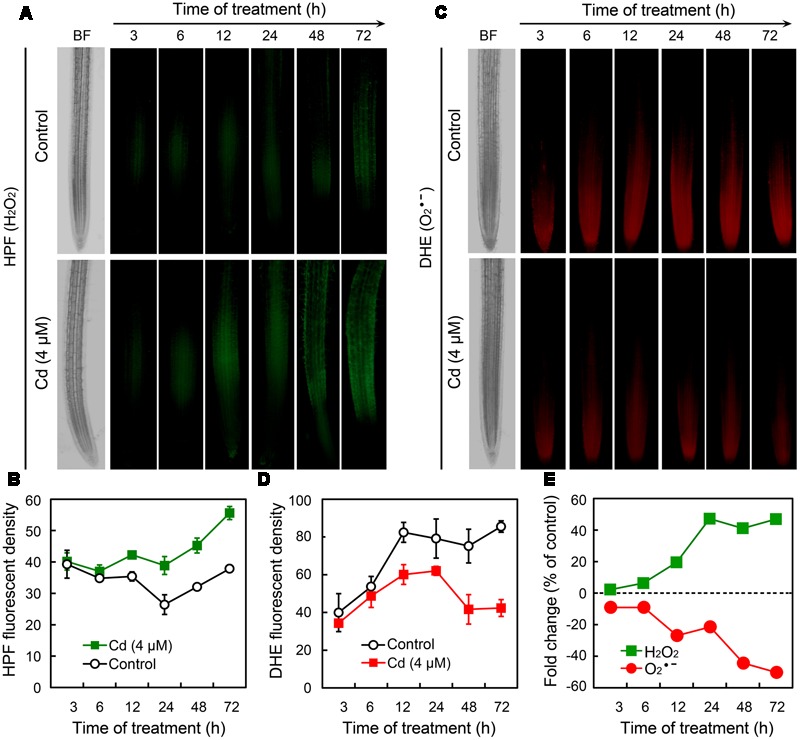
**Time-course changes of endogenous H_2_O_2_ and $O_{2}^{•–}$ in the root tip of *B. rapa* under the treatment of Cd at 4 μM. (A–D)** The roots of seedlings were exposed to 0 (control) and 4 μM of CdCl_2_ for 3, 6, 12, 24, 48, and 72 h, respectively. Then the roots were loaded with HPF or DHE to obtain HPF fluorescent image **(A)**, HPF fluorescent density **(B)**, DHE fluorescent image **(C)**, and DHE fluorescent density **(D)**. **(E)** CdCl_2_ (4 μM)-induced fold changes of HPF and DHE fluorescent density in root tip as compared to the control groups.

To further ascertain the responses of ROS in root tips under Cd exposure, we monitored the changes of H_2_O_2_ and $O_{2}^{•–}$ in a time-course experiment. Compared to the control group, treatment with Cd at 4 μM led to the significant increase in H_2_O_2_ after 12 h (**Figures 3A,B**). In contrast, $O_{2}^{•–}$ began to decrease remarkably in root tip treated with 4 μM of Cd after 12 h (**Figures 3C,D**). The changing patterns of H_2_O_2_ and $O_{2}^{•–}$ were also indicated by the fold change with respect to control (**Figure 3E**). These results demonstrated that treatment with Cd at 4 μM disturbed ROS balance by decreasing $O_{2}^{•–}$ and increasing H_2_O_2_ in the root tip of *B. rapa*.

### The Altered ROS Balance was Closely Linked to the Inhibition of Root Elongation Under Cd (4 μM) Exposure

**FIGURE 4:**
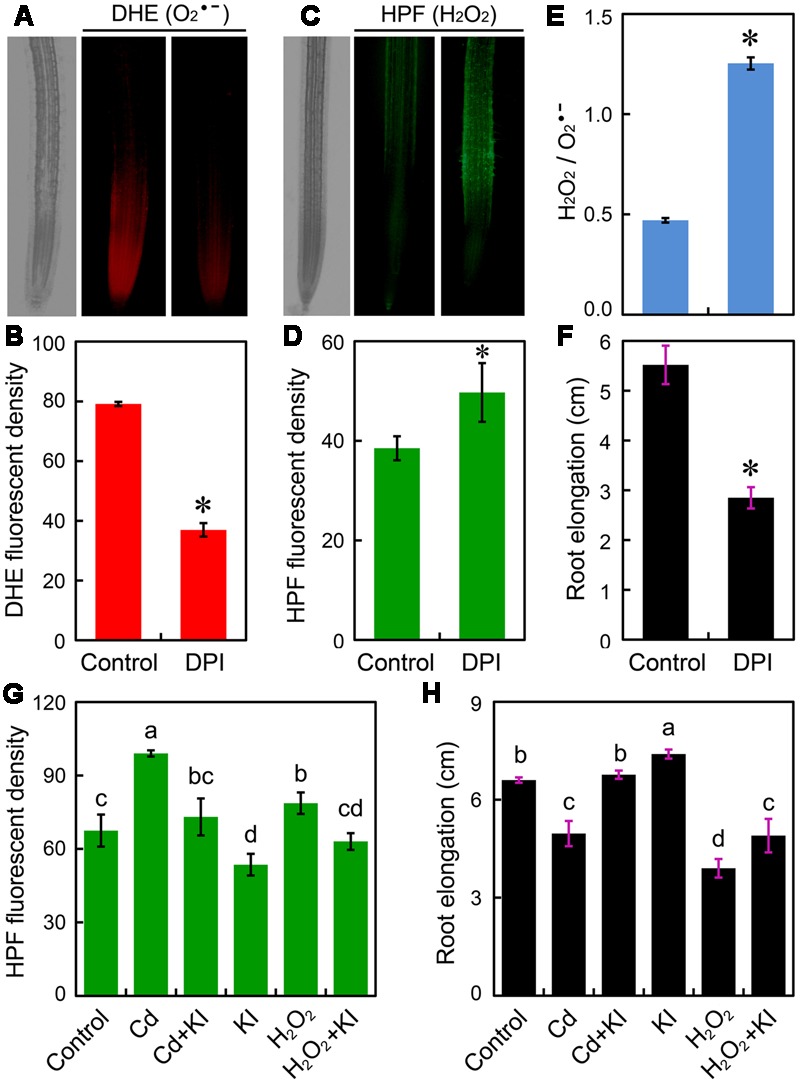
**Effect of reactive oxygen species (ROS) scavengers on root elongation and ROS content in the root tip of *B. rapa*. (A–F)** The roots of seedlings were exposed to 0 (control) and 0.5 μM of DPI for 72 h. Then DHE fluorescent image **(A)**, DHE fluorescent density **(B)**, HPF fluorescent image **(C)**, HPF fluorescent density **(D)**, HPF fluorescent density (H_2_O_2_)/DHE fluorescent density ($O_{2}^{•–}$) **(E)**, and root elongation **(F)** were determined. **(G,H)** The fluorescent density of HPF **(G)** and root elongation **(F)** were measured when the roots of seedlings were exposed to distilled water (control), CdCl_2_ (4 μM), CdCl_2_ (4 μM)+KI (50 μM), KI (50 μM), H_2_O_2_ (1.8 mM), and H_2_O_2_ (1.8 mM)+KI (50 μM) for 72 h. *Asterisk* (^∗^) indicates that mean values of three replicates are significantly different between the treatment and control (*P* < 0.05) in **(B)** and **(D–F)**. The mean values of three replicates followed by different letters indicate significance of difference between the treatments (*P* < 0.05, ANOVA, LSD) in **(G,H)**.

Since Cd at 4 μM differentially regulated H_2_O_2_ and $O_{2}^{•–}$ without inducing oxidative injury in root tip, we wondered whether the altered ROS balance was associated with the Cd-induced growth retardation of root. To confirm the role of $O_{2}^{•–}$ in the positive regulation of root elongation, DPI was applied to inhibit NADPH oxidase that is one of the major source of $O_{2}^{•–}$ generation in plant cells. Treatment with DPI significantly decreased endogenous $O_{2}^{•–}$ content in root tips (**Figures 4A,B**), coinciding with the significant increase in H_2_O_2_ and the ratio of H_2_O_2_/$O_{2}^{•–}$ as well as the remarkable decrease in root elongation (**Figures 4C–F**). Exogenous application of H_2_O_2_ resulted in considerable increase in endogenous H_2_O_2_ and significant decrease in root elongation, which was similar to the action of treatment with Cd at 4 μM (**Figures 4G,H**). Treatment with KI (H_2_O_2_ scavenger) was able to decrease endogenous H_2_O_2_ content and to promote root elongation in the presence of Cd (4 μM) or not (**Figures 4G,H**). Notably, scavenging excessive H_2_O_2_ by KI led to the recovery of growth phenotype under treatment of Cd at 4 μM (**Figures 4G,H**). These results evidenced that the inhibition of root elongation induced by Cd at 4 μM may result from the decrease in $O_{2}^{•–}$ and the increase in H_2_O_2_ in root tip.

### Endogenous H_2_S was Involved in the Differential Regulation of H_2_O_2_ and $O_{2}^{•–}$ in Cd-Treated Root

**FIGURE 5:**
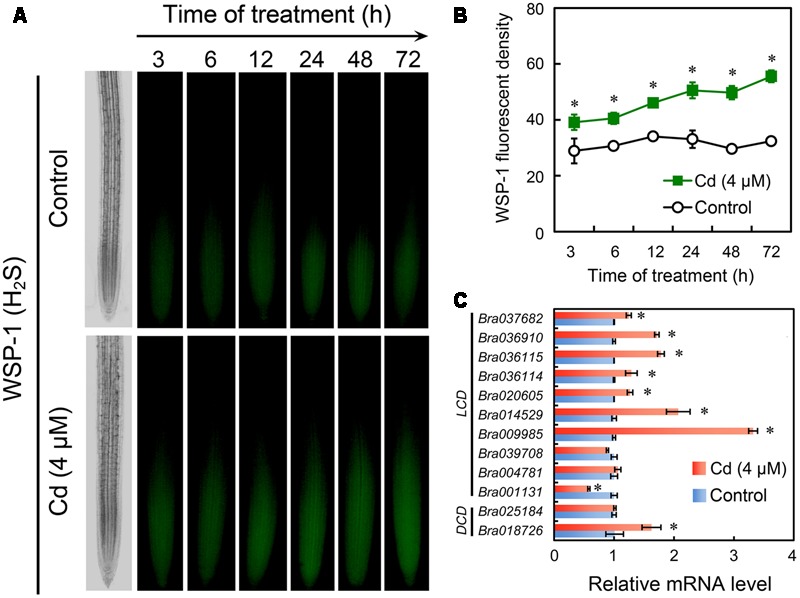
**Effect of Cd (4 μM) on the content of endogenous H_2_S and the expression of *LCDs*/*DCDs* in the root tip of *B. rapa*. (A,B)** The roots of seedlings were exposed to 0 (control) and 4 μM of CdCl_2_ for 3, 6, 12, 24, 48, and 72 h, respectively. Then the roots were loaded with WSP-1 to obtain WSP-1 fluorescent image **(A)** and WSP-1 fluorescent density **(B)**. **(C)** The roots of seedlings were exposed to 0 (control) and 4 μM of CdCl_2_ for 72 h. Then the root tips were harvested for RNA extraction and real-time PCR analysis for the expression levels of *LCDs* and *DCDs*. *Actin* was used for cDNA normalization. *Asterisk* indicates that mean values of three replicates are significantly different between treatments and control (*P* < 0.05) in **(B,C)**.

Specific fluorescent detection of H_2_S has been suggested as a promising method to localize and quantify H_2_S precisely in cells because the in-tube assay of H_2_S content in tissues always leads to unavoidable losses and failure to the cellular compartmentalization of H_2_S (Hancock and Whiteman, 2016). In the present study, the endogenous H_2_S in root tip was selectively tracked *in vivo* by fluorescent probe WSP-1. H_2_S preferred to accumulate in EZ in root tip (**Figure 5A**). In a time-course test up to 72 h, treatment with Cd at 4 μM resulted in the continuous increase in endogenous H_2_S level in root tip as compared to the control group (**Figure 5B**). In our previous study, the *in silico* analysis suggested that there were ten *LCD* orthologues and two *DCD* orthologues in the genome of *B. rapa* (Chen et al., 2014). Transcriptional analysis suggested that treatment with Cd at 4 μM induced significant up-regulation of the expression of seven *LCDs* (*Bra037682, Bra036910, Bra036115, Bra036114, Bra020605, Bra014529*, and *Bra009985*) and one *DCD* (*Bra018726*) in the root tip of *B. rapa*. The expression of two *LCDs* (*Bra039708* and *Bra004781*) and one *DCD* (*Bra025184*) were not impacted significantly by treatment with 4 μM of Cd. The expression of only one *LCD* (*Bra001131*) was down-regulated by treatment with 4 μM of Cd (**Figure 5C**). These results suggested treatment with Cd at 4 μM stimulated the generation of endogenous H_2_S in root tip, which may resulted from the extensive up-regulation of *LCDs* and *DCDs*.

**FIGURE 6:**
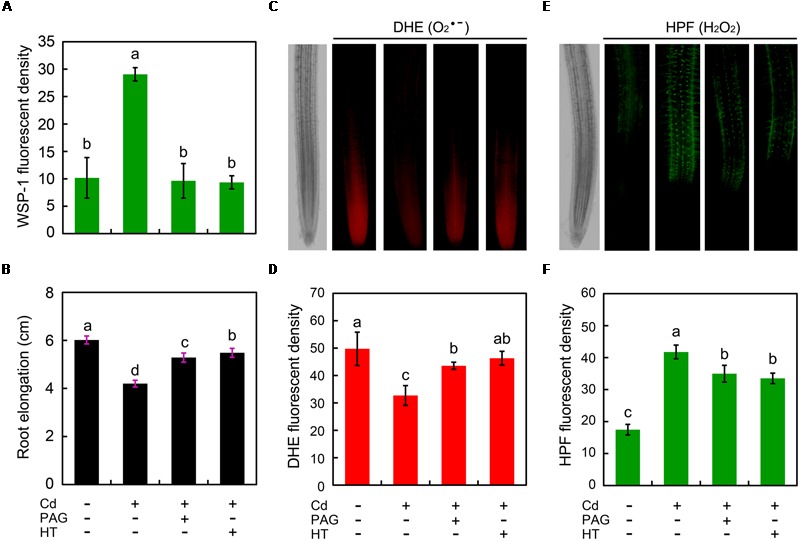
**Effect of PAG (DL-propargylglycine) and HT (hypotaurine) on root elongation as well as the content of endogenous H_2_S, H_2_O_2_, and $O_{2}^{•–}$ in the root tip of *B. rapa* under treatment of Cd (4 μM).** The roots of seedlings were exposed to distilled water (control), CdCl_2_ (4 μM), CdCl_2_ (4 μM)+PAG (0.05 μM), and CdCl_2_ (4 μM)+HT (3 μM) for 72 h. Then the roots were loaded with WSP-1 for the quantification of WSP-1 fluorescent density **(A)**. The root elongation was measured **(B)**. The roots were loaded with DHE to obtain DHE fluorescent image **(C)** and DHE fluorescent density **(D)**. The roots were loaded with HPF to obtain HPF fluorescent image **(E)** and HPF fluorescent density **(F)**. The mean values of three replicates followed by different letters indicate significance of difference between the treatments (*P* < 0.05, ANOVA, LSD).

To investigate the possible role of endogenous H_2_S in the regulation of root growth and ROS balance in Cd-treated root, PAG (endogenous H_2_S biosynthesis inhibitor) and HT (H_2_S scavenger) were added to the treatment solution, respectively. The addition of PAG or HT reversed the stimulatory effect of Cd (4 μM) on endogenous H_2_S (**Figure 6A**), coinciding with the recovery of root elongation upon 4 μM of Cd (**Figure 6B**). Intriguingly, the addition of PAG or HT was able to significantly increase the endogenous $O_{2}^{•–}$ level in root tip under 4 μM of Cd treatment with (**Figures 6C,D**). In addition, PAG or HT remarkably inhibited the increase in endogenous H_2_O_2_ level in Cd (4 μM)-treated root tip (**Figures 6E,F**). These results revealed that the endogenous H_2_S mediated Cd (4 μM)-induced retardation of root elongation by altering the balance between H_2_O_2_ and $O_{2}^{•–}$ in the root tip of *B. rapa*.

### Endogenous H_2_S was Involved in the Regulation of Br_UPB1 and Its Downstream Events in Cd-Treated Root

**FIGURE 7:**
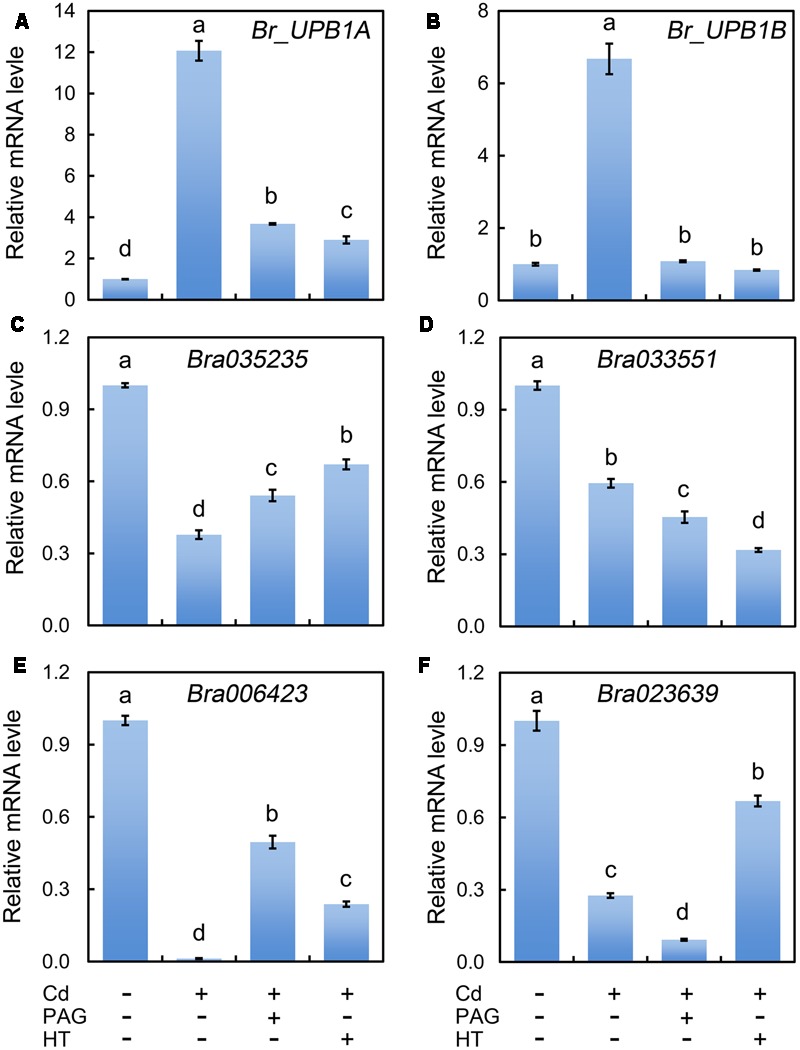
**Effect of PAG and HT on the expression of *Br_UPB1s* and peroxidase genes in the root tip of *B. rapa* under treatment of Cd (4 μM).** The roots of seedlings were exposed to distilled water (control), CdCl_2_ (4 μM), CdCl_2_ (4 μM)+PAG (0.05 μM), and CdCl_2_ (4 μM)+HT (3 μM) for 72 h. Then the root tips were harvested for RNA extraction and real-time PCR analysis of the expression of *Br_UPB1s* (*Br_UPB1A* and *Br_UPB1B*) **(A,B)** and peroxidase genes (*Bra035235, Bra033551, Bra006423, Bra023639*) **(C–F)**. *Actin* was used for cDNA normalization. The mean values of three replicates followed by different letters indicate significance of difference between the treatments (*P* < 0.05, ANOVA, LSD).

During the root elongation in *Arabidopsis*, UPB1 act as a transcriptional factor to repress the expression of several *PODs* for the further controlling of the balance between H_2_O_2_ and $O_{2}^{•–}$ (Tsukagoshi et al., 2010). Therefore, we needed to know whether *UPB1* could be regulated by Cd. Based on BLAST search against *AtUPB1* (*At2g47270*), two homologues (*Bra004465, Br_UPB1A*; *Bra021395, Br_UPB1B*) were retrieved from the genome of *B. rapa*. The multi-alignment of deduced amino acid sequences indicated that both Br_UPB1A and Br_UPB1B with conserved bHLH domains shared high similarity with At_UPB1 (Supplementary Figure 3). The expression of both *Br_UPB1A* and *Br_UPB1B* were improved remarkably under the treatment of Cd at 4 μM remarkably as compared to control group, both of which were inhibited by the addition of PAG or HT (**Figures 7A,B**).

The *Arabidopsis* bHLH transcript factor family includes two groups, DNA-binders and non-DNA-binders, based on the DNA-binding capacity. At_UPB1 belongs to non-DNA-binder without E-box DNA binding capacity based on the absence of amino acid E41 and/or R44 in the “Basic” domain (Toledo-Ortiz et al., 2003). The similar feature was also found in Br_UPB1A and Br_UPB1B (Supplementary Figure 3). The mechanism for non-DNA-binding bHLH on the regulation of target genes is still elusive, the ChIP-chip study indicated that At_UPB1 negatively regulated root elongation by directly suppressing the expression of several peroxidase genes (*At4g11290, Per39*; *At4g16270, Per40*; *At5g17820, Per57*) (Tsukagoshi et al., 2010). In the present study, we retrieved the homologues of these *Arabidopsis* peroxidases from the genome of *B. rapa*. *Bra035235* and *Bra033551* were homologues of *At4g11290* and *At4g16270*, respectively. Both *Bra023639* and *Bra006423* were the homologues of *At5g17820* (Supplementary Figures 4 and 5). As expected, the expression of all these four peroxidase genes were inhibited pronouncedly in Cd (4 μM)-treated roots compared to the control samples (**Figures 7C–F**). Notably, the expression of *Bra006423* was almost completely suppressed by Cd (4 μM) treatment (**Figure 7E**). As compared to Cd treatment alone, treatment with PAG+Cd or HT+Cd significantly enhanced the transcriptional level of *Bra035235* and *Bra006423* (**Figures 7C,E**). These results suggested that endogenous H_2_S up-regulated the expression of *Br_UPB1*, which may further suppressed the expression of two peroxidase genes (*Bra035235* and *Bra006423*) in Cd (4 μM)-treated roots.

### Hierarchical Cluster Analysis of the Interaction of H_2_S and ROS in Roots Exposed to Cd

**FIGURE 8:**
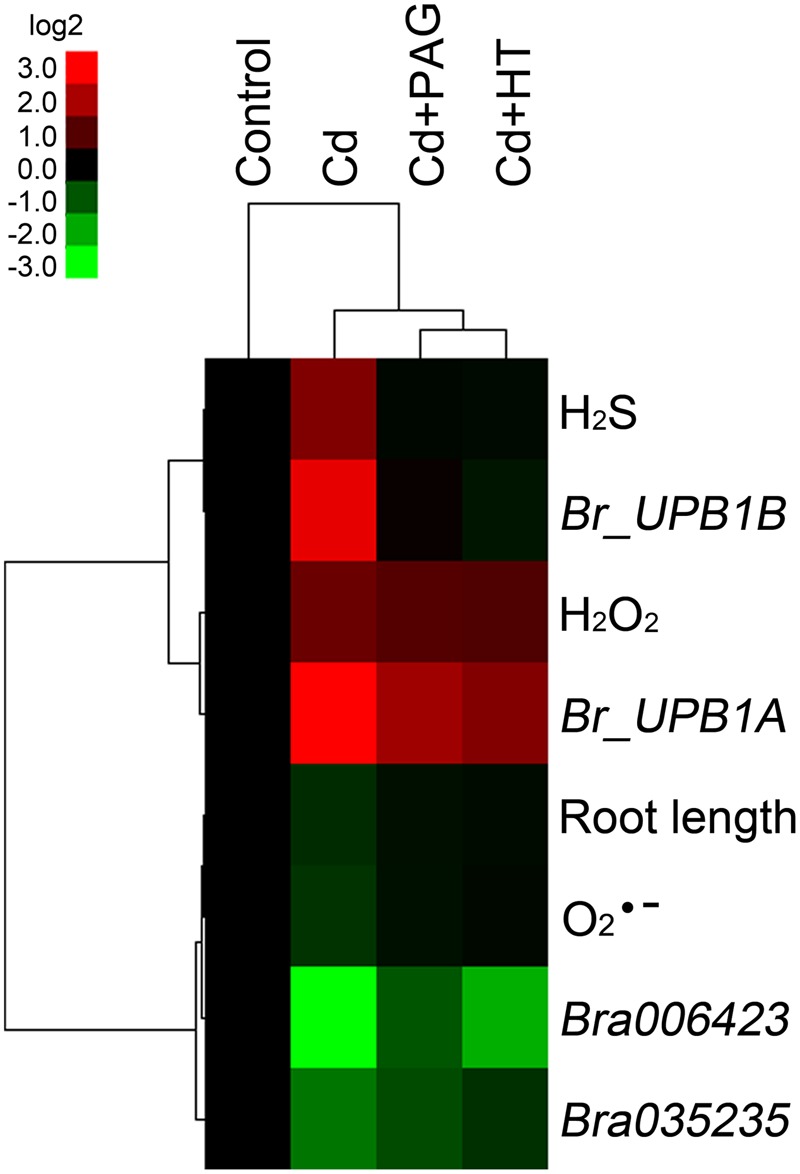
**Hierarchical cluster analysis of the effects of PAG and HT on physiological responses of *B. rapa* to Cd (4 μM) treatment.** The relative data of endogenous H_2_S, H_2_O_2_, $O_{2}^{•–}$ content (presented as specific fluorescent density), root elongation, the expression level of *Br_UPB1A, Br_UPB1B, Bra006423*, and *Bra035235* in the root tip of *B. rapa* with different treatments, were selected for cluster analysis. All the data were presented as relative fold change respect to the control. The cluster color bar was shown as log_2_ fold change. The tree was generated by using Cluster 3.0 and Java Treeview as described in Section “Materials and Methods”.

Based on the obtained data of root length, endogenous $O_{2}^{•–}$, H_2_O_2_, H_2_S content, and the expression of *Br_UPB1A, Br_UPB1B, Bra006423*, and *Bra035235* in roots upon the treatments of different chemicals (**Figures 6** and **7**), hierarchical clustering was performed to analyze the relationship among biochemical parameters or different treatments (**Figure 8**). Treatment with endogenous H_2_S biosynthesis inhibitor (PAG) or H_2_S scavenger (HT) blocked Cd-induced H_2_S accumulation, and showed attenuated effects on Cd-induced changes in other parameters (**Figure 8**), suggesting that H_2_S mediated Cd-induced phytotoxcity. All the parameters are classified to two groups. H_2_S, H_2_O_2_, *Br_UPB1A*, and *Br_UPB1B* were stimulated by Cd treatment, indicating that these parameters contributed to Cd toxicity. However, the root length, $O_{2}^{•–}$, *Bra006423*, and *Bra035235* were repressed by Cd treatment, suggesting that these parameters were negatively regulated by Cd exposure (**Figure 8**).

## Discussion

Cd is able to induce the increase in H_2_O_2_ and the decrease in $O_{2}^{•–}$ in the roots of *G. max* and *C. sativus* (Heyno et al., 2008). However, how Cd differentially regulates H_2_O_2_ and $O_{2}^{•–}$ in plant cells remains unclear. H_2_S is an important signaling molecule regulating plant intrinsic physiology (Jin and Pei, 2015). Here we provide evidences that Cd induces the disturbance between H_2_O_2_ and $O_{2}^{•–}$ as well as the subsequent growth retard in the roots of *B. rapa*, which is dependent on the expression of *Br_UPB1* regulated by endogenous H_2_S.

Cadmium stress frequently induces the accumulation of both H_2_O_2_ and $O_{2}^{•–}$, leading to the occurrence of oxidative damage (Pérez-Chaca et al., 2014). Here we also found that Cd at relatively high concentration (16 μM) resulted in the accumulation of both H_2_O_2_ and $O_{2}^{•–}$ in the root tip of *B. rapa*, which was confirmed by the subsequent occurrence of oxidative injury and cell death. However, Cd at low concentration (4 μM) was able to inhibit root elongation without inducing oxidative injury and cell death, coinciding with the increase in H_2_O_2_ and decrease in $O_{2}^{•–}$ in root tip. These results promoted us to think about the signaling roles of ROS in the regulation of root growth under Cd stress, rather than the induction of oxidative stress.

Root tip is the important expansion zone responsible for root elongation (Dupuy et al., 2010). In the present study, H_2_O_2_ and $O_{2}^{•–}$ were detected to be mainly located in DZ and EZ+MZ of *B. rapa* root tip, respectively, which is similar with the distribution pattern of H_2_O_2_ and $O_{2}^{•–}$ in the root tip of *Arabidopsis* (Dunand et al., 2007). Scavenging H_2_O_2_ with KI promoted root elongation of *B. rapa* under Cd (4 μM) treatment or normal conditions, advocating a negative role for H_2_O_2_ in the regulation of root elongation. NADPH oxidase encoded by *rbohs* (*respiratory burst oxidative homologs*) has been suggested as a major source for $O_{2}^{•–}$ generation in plant cells (Suzuki et al., 2011). Treatment with DPI, a NADPH oxidase inhibitor, inhibited $O_{2}^{•–}$ generation and root elongation, akin to the action of Cd (4 μM) treatment. DPI treatment also stimulated H_2_O_2_ generation in root tip, leading to the increase in the ratio of H_2_O_2_/$O_{2}^{•–}$. Thus, it can be speculated that the balance between H_2_O_2_ and $O_{2}^{•–}$ is vital for root elongation under Cd stress. In addition, DPI treatment may affect other proteins activities besides NADPH oxidase because DPI is a kind of general inhibitor of flavin-containing enzymes but not a specific inhibitor to NADPH oxidase (Bolwell, 1999; Moulton et al., 2000). Therefore, genetic evidences are needed to identify the role of NADPH oxidase-derived $O_{2}^{•–}$ in the regulation of root elongation upon Cd exposure.

In *Arabidopsis*, over-expression of *UPB1* inhibited root elongation by increasing H_2_O_2_ and decreasing $O_{2}^{•–}$ in root tip, while the insertional mutation (*upb1-1*) showed adverse effects (Tsukagoshi et al., 2010). And the balance between H_2_O_2_ and $O_{2}^{•–}$ maintained by *UPB1* seems to regulate root elongation by modulating the onset of cell differentiation but not oxidative injury in root tip. Root cells stop proliferating and start to elongate when the ratio of $O_{2}^{•–}$/H_2_O_2_ reaches a proper level (Tsukagoshi et al., 2010). Here we found that Cd (4 μM) treatment remarkably up-regulated the expression of two *UPB1* homologues (*Br_UPB1A* and *Br_UPB1B*) in the root tip of *B. rapa*, which may explain the downstream observation of ROS alteration and root inhibition without showing oxidative injury. Peroxidase is capable of scavenging H_2_O_2_ by catalyzing H_2_O_2_ to H_2_O. In the root tip of *Arabidopsis*, genetic evidences suggested that UPB1 promoted H_2_O_2_ generation by negatively regulating the expression of several peroxidase genes (Tsukagoshi et al., 2010). In the present study, Cd (4 μM) treatment resulted in the down-regulation of four peroxidase gene homologues in the root tip of *B. rapa*, leading to the increase in H_2_O_2_. For the decrease in $O_{2}^{•–}$ observed in this study, one possible reason is the regulation of *rbohs*. It has been reported that Cd treatment inhibited NADPH oxidase activity and $O_{2}^{•–}$ generation *in vivo* in *Helianthus annuus* (Groppa et al., 2012). Although the functional redundancy for the maintenance of root meristem may exist among different *rboh* genes, the loss of *upb1* function mutation resulted in the up-regulation of at least five *rbohs* in *Arabidopsis* (Tsukagoshi et al., 2010). Therefore, it is possible that Cd (4 μM) treatment inhibit $O_{2}^{•–}$ generation by inducing the expression of *Br_UPB1s* that may further lead to the repression of *rbohs*. In addition, it has been suggested that $O_{2}^{•–}$ generation might be driven by the consumption of H_2_O_2_ by peroxidase in *Arabidopsis upb1-1* mutant (Tsukagoshi et al., 2010). Our present data demonstrated that H_2_O_2_ generation was promoted by decreasing NADPH oxidase-dependent $O_{2}^{•–}$ generation in the root tip of *B. rapa*. Therefore, it is interesting to further investigate the mechanism for the modulation between H_2_O_2_ and $O_{2}^{•–}$ by each other during *UPB1*-modulated root elongation under Cd stress or normal growth conditions.

Hydrogen sulfide has been considered as an important node connecting multiple signaling pathways in plants (Jin and Pei, 2015). H_2_S is able to scavenge ROS by enhancing anti-oxidative capacity in plants under intense environmental stimuli (Hancock and Whiteman, 2015, 2016), but here we found a precise control of the balance between H_2_O_2_ and $O_{2}^{•–}$ by endogenous H_2_S in the root tip of *B. rapa* under relatively slighter Cd stimulus. In our current results, three lines of evidence indicated that Cd (4 μM) treatment resulted in *Br_UPB1s*- modulated ROS balance and root inhibition by triggering endogenous H_2_S generation in root tip. First, Cd (4 μM) treatment resulted in the increase in endogenous H_2_S by up-regulating the expression of *LCDs* and *DCD*. Second, PAG or HT led to the decrease in endogenous H_2_S level, which further reversed Cd (4 μM)-induced changes of the expression level of *Br_UPB1s* and its two possible target peroxidase genes. Third, the decrease in endogenous H_2_S by either PAG or HT resulted in the recovery from Cd (4 μM)-induced ROS balance alteration and root inhibition. LCD/DCD-dependent H_2_S generation has been found in *Medicago sativa, Arabidopsis*, and *B. rapa* under Cd exposure at high concentration (Cui et al., 2014; Qiao et al., 2015, 2016; Zhang et al., 2015). And their reports suggest that H_2_S acts as a cytoprotectant scavenging Cd-induced over-generation of H_2_O_2_, $O_{2}^{•–}$, and total ROS in plants. However, our present results revealed that LCD/DCD-dependent generation of endogenous H_2_S disturbed the balance between H_2_O_2_ and $O_{2}^{•–}$, which further contributed the phytotoxicity induced by Cd at low concentration. Therefore, it can be proposed that H_2_S triggers distinct ROS signaling pathways in plant cells in response to different levels of Cd exposure. In the present study, pharmacological results suggested that endogenous H_2_S mediated Cd (4 μM)-arrested root elongation probably through the stimulation of *Br_UPB1s*-regulated cell proliferation in root tip. Intriguingly, tumor-derived endogenous H_2_S stimulates cell proliferation in colon cancer by regulating Akt kinase and ERK (extracellular signal-regulated kinase) signaling pathways in mammalian cells (Cai et al., 2010; Szabo et al., 2013; Szabo and Hellmich, 2013). Further study on the difference of H_2_S-regulated cell cycle between plants and mammals would help our understanding of the mechanisms for H_2_S to modulate Cd adaption in plants.

In addition to H_2_S, NO plays important role in the regulation of root growth. The crosstalk between H_2_S and NO has been suggested to be involved in the modulation of plant adaption to Cd stress (Li et al., 2012; Shi et al., 2014) and root development (Zhang et al., 2009; Li et al., 2014). It has been documented that Cd inhibits meristem growth in the root tip of *Arabidopsis*. The suppression of Cd-induced NO accumulation compromised Cd-induced root meristem development, indicating that endogenous NO mediates the inhibition of root meristem growth under Cd exposure (Yuan and Huang, 2016). The interaction among H_2_S, NO, and ROS exists extensively in both plants and mammals (Hancock and Whiteman, 2016). Therefore, whether NO functions in H_2_S-regulated ROS balance in the modulation of Cd-inhibited meristem growth needs to be investigated further.

**FIGURE 9:**
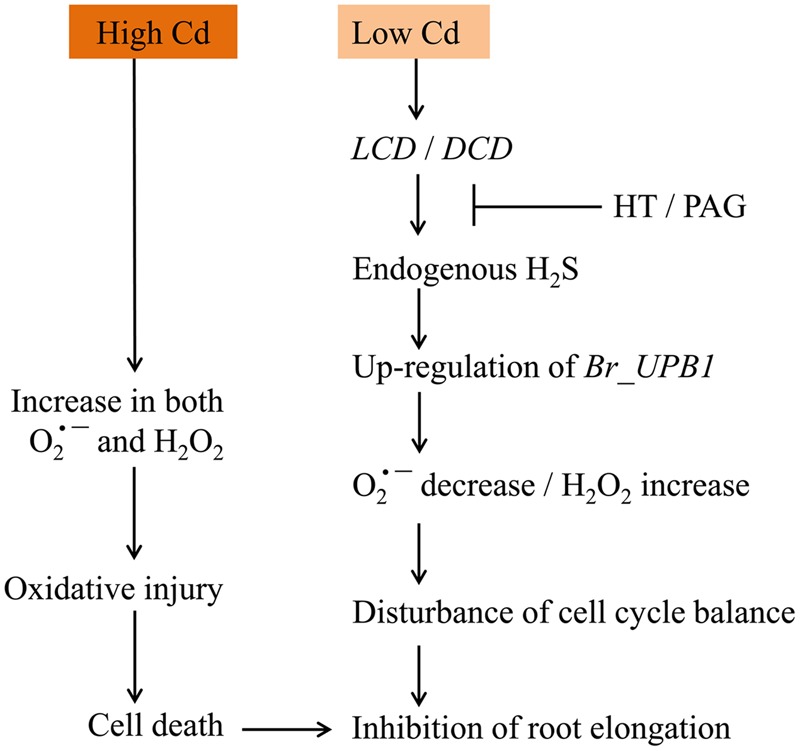
**The schematic model for Cd-induced root inhibition by differential regulation of ROS balance**.

In sum, a working model was obtained based on our results (**Figure 9**). Cd exposure at low concentration led to *LCDs*/*DCD*-dependent generation of endogenous H_2_S, which further induced the up-regulation of *Br_UPB1s* in root tip. Then the decrease in $O_{2}^{•–}$ and increase in H_2_O_2_ were triggered, leading to the inhibition of root elongation by probably modulating cell proliferation in root tip. However, Cd exposure at high concentration directly resulted in the increase in both H_2_O_2_ and $O_{2}^{•–}$, leading to the occurrence of oxidative injury following by cell death and root growth inhibition. This study not only sheds new light on the regulatory role of H_2_S in modulating ROS signaling, but also extends our knowledge to understand the mechanism for plant adaptations to Cd stress.

## Author Contributions

JC and LY designed the experiments. WL, CX, JS, and ZS performed the experiments. JC and WL analyzed the data. JC and MX contributed to reagents and materials. JC and LY wrote the paper.

## Conflict of Interest Statement

The authors declare that the research was conducted in the absence of any commercial or financial relationships that could be construed as a potential conflict of interest.

The reviewer JG declared a shared affiliation, though no other collaboration, with one of the authors XM to the handling Editor, who ensured that the process nevertheless met the standards of a fair and objective review.

## Footnotes

1 http://bonsai.hgc.jp/mdehoon/software/cluster/

2 https://sourceforge.net/projects/jtreeview/
