# Supplementary material for: Cadmium Disrupts the Balance between Hydrogen Peroxide and Superoxide Radical by Regulating Endogenous Hydrogen Sulfide in the Root Tip of Brassica rapa
Source: Front Plant Sci. 2017 Feb 21;8:232. doi: 10.3389/fpls.2017.00232 (PMC5318417; doi:10.3389/fpls.2017.00232)
Supplement: Supplementary file 1 [file Presentation_1.pdf]

Supplementary Material

Cadmium disrupts the balance between hydrogen peroxide and superoxide radical by regulating endogenous hydrogen sulfide in the root tip of *Brassica rapa*

Wenjing Lv, Lifei Yang, Cunfa Xu, Zhiqi Shi, Jinsong Shao, Ming Xian, Jian Chen*

*** Correspondence:** jacksonchen206@gamil.com (J. C.)

1 Supplementary materials and methods

About 1 cm root sections from root tips were collected for the determination of MDA content. A MDA detection kit (A003; Nanjing Jiancheng Bioengineering Institute, Nanjing, China) was selected to measure the MDA level based on the spectrophotometric determination of the reaction between MDA and TBA (1,3-diethyl-2-thiobarbituric acid) assisted by TCA (trichloroacetic acid) (Yang et al., 2012).

Histochemical detection of endogenous $O_2^{\bullet -}$(superoxide radical) in root tip was performed by using NBT (nitro-blue tetrazolium) staining as described by Frahry and Schopfer (2001). The roots of seedlings after treatment were transferred to 10 mM Na-citrate buffer (pH 6.0) containing 6 mM NBT under light at 25 ℃ for 20 min, and then the roots were rinsed with distilled water for three times, which allowed the dark blue insoluble formazan compound (by reaction of NBT with $O_2^{\bullet -}$) inside of roots to be clearly visualized and photographed.

Histochemical detection of endogenous H_2O_2 (hydrogen peroxide) in root tip was performed by using DAB (3,3-diaminobenzidine) staining as described by Nguyen et al (2013). The roots of seedlings after treatment were transferred to 0.1% (w/v) of DAB-HCl solution (pH 3.8) for the incubation of 20 min. Then the roots were rinsed with distilled water for three times, which allowed deep brown polymerization product (reaction of DAB and H_2O_2) to be clearly visualized and photographed.

For the measurement of H_2O_2 content, approximate 0.1 g of root tip samples were homogenized in 1.5 ml phosphate buffer (50 mM, pH 6.5) containing 1 mM of hydroxylamine. After centrifugation at 10000 g for 10 min, 0.5 ml supernatant was mixed with 1.5 ml 20% (v/v) H_2SO_4, containing 0.1% (v/v) $TiCl_4$. After thorough mixing, the mixture was centrifuged at 10000 g for 10 min. Then the absorbance of the supernatant was measured at 410 nm. The content of H_2O_2 was calculated using an extinction coefficient of 0.28 /μM/cm (Jana and Choudhuri, 1982).

For the measurement of $O_2^{\bullet -}$content, approximate 0.1 g of root tip samples were homogenized in 1.5 ml phosphate buffer (50 mM, pH 7.8). After centrifugation at 5000 g for 10 min at 4 ℃, 0.5 ml of supernatant was mixed with 1 ml of hydroxylamine hydrochlorides (1 mM) and 0.5 ml of phosphate buffer (pH 7.8) for the reaction of 1 h at 25 ℃. Then 1 ml of *p*-aminobenzene sulfonic acid (17 mM)

and 1 ml of α-naphthylamine (7 mM) were added for another reaction of 20 min at 25 ℃. The absorbance of the mixture nm was determined at 530 nm. A standard curve of $NaNO_2$ was prepared to calculate the content of $O_2^{\bullet -}$ based on fresh weight (Chen et al., 2013).

The *Arabidopsis* gene sequences used were retrieved from TAIR (The *Arabidopsis* information resources) (http://www.arabidopsis.org/). The Arabidopsis sequences were used as baits for BLAST search in the genome of *Brassica rapa* from BRAD (*Brassica* database) (http://brassicadb.org/brad/index.php). The multi-alignment of deduced amino acid sequences was performed with Clustal X 2.0 and DNAMAN 5.2.2 (Larkin et al., 2007). The conserved domains in amino acid sequences were predicted and analyzed by using online tool SMART (Simple Modular Architecture Research Tool) (http://smart.embl-heidelberg.de/) (Letunic et al., 2012).

2 Supplementary Figures and Tables

2.1 Supplementary Figures

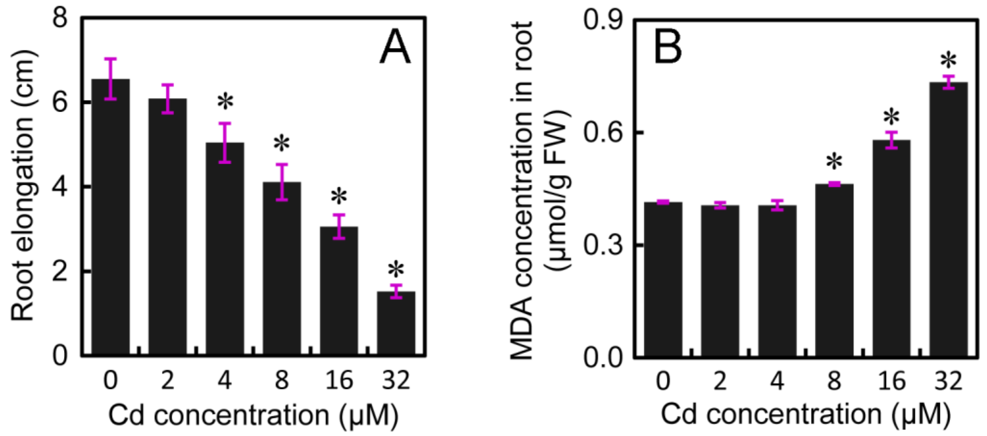

Supplementary Figure 1 | Effect of Cd treatment on root elongation and MDA content in the root tip of *B. rapa*. The roots of seedlings were exposed to 0, 2, 4, 8, 16 and 32 μM of $CdCl_2$ for 72 h. Then the root length was measured (A). The root tips after treatment were collected for the measurement of MDA content (B). *Asterisk* (*) indicates that mean values of three replicates are significantly different between the treatment and control (P<0.05).

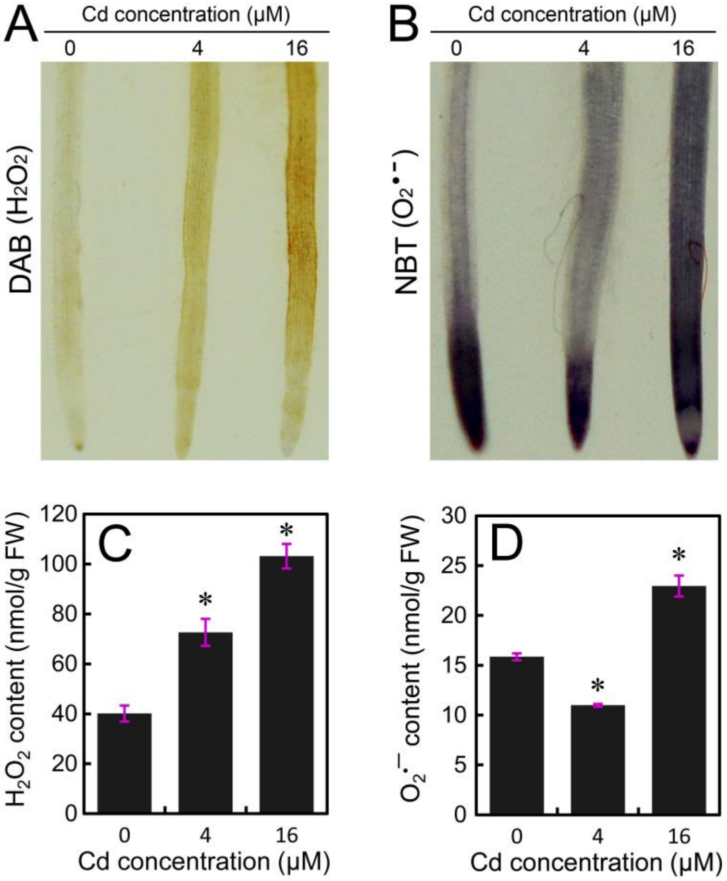

Supplementary Figure 2 | Effect of Cd treatment on H_2O_2 and $O_2^{\bullet-}$ in the root tip of *B. rapa*. The roots of seedlings were exposed to 0, 4, and 16 μM of $CdCl_2$ for 72 h. Then the roots were stained with DAB indicating H_2O_2 (A) and NBT indicating $O_2^{\bullet-}$(B), respectively. The root tips after treatment were collected for the measurement of H_2O_2 content (C) and $O_2^{\bullet-}$content (D), respectively. *Asterisk* (*) in (C) and (D) indicates that mean values of three replicates are significantly different between the treatment and control (P<0.05).

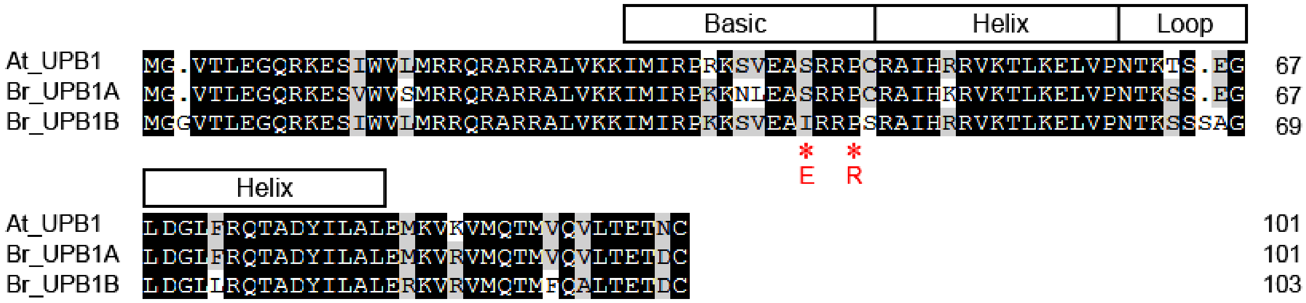

Supplementary Figure 3 | Multi-alignment of amino acid sequences of At_UPB1 (At2g47270), Br_UPB1A (Bra004465), and Br_UPB1B (Bra021395). Four conserved domains (Basic-Helix-Loop-Helix) were indicated as *black box*. *Asterisk* indicates the absence of amino acid E and R, both of which are indispensable for DNA-binding capacity of the protein. *Dark shading* with *white letters* and *gray shading* with *black letters* reveal 100% and 75% sequence similarity, respectively.

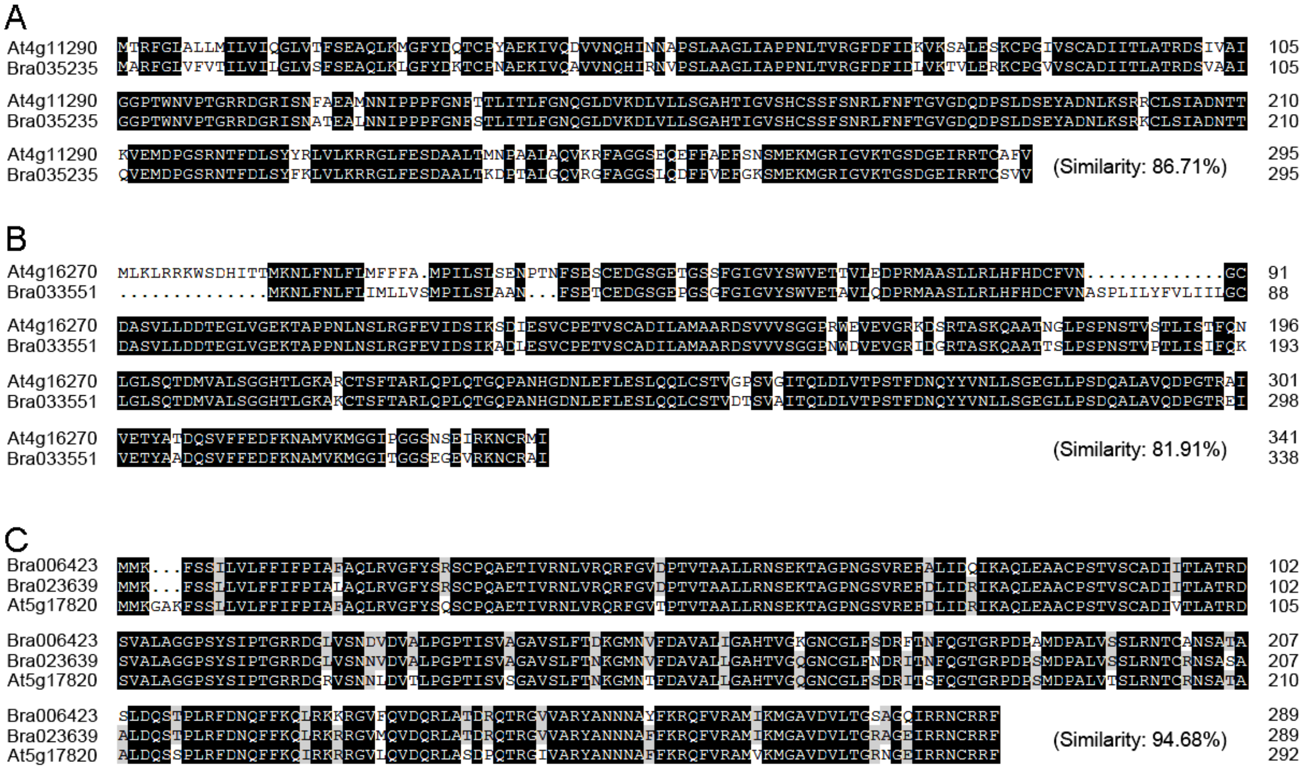

Supplementary Figure 4 | Multi-alignment of amino acid sequences of peroxidase from *Arabidopsis* and *B. rapa*. (A) Alignment between *Arabidopsis* peroxidase Per39 (At4g11290) and its homologues (Bra035235) in *B. rapa*. **(B)** Alignment between *Arabidopsis* peroxidase Per40 (At4g16270) and its homologues (Bra033551) in *B. rapa*. **(C)** Multi-alignment among *Arabidopsis* peroxidase Per57 (At4g17820) and its two homologues (Bra036423 and Bra023639) in *B. rapa*. *Dark shading* with *white letters* and *gray shading* with *black letters* reveal 100% and 75% sequence similarity, respectively.

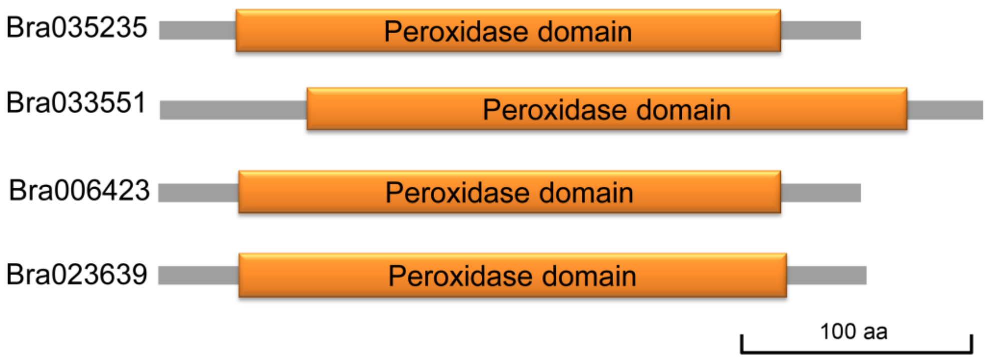

Supplementary Figure 5 | The location of peroxidase domain in four *B. rapa* peroxidases (Bra035235, Bra033551, Bra036423, and Bra023639). The sequences were analyzed by SMART. The typical and conserved peroxidase domain was indicated as orange box. Bar indicated 100 amino acids (aa).

2.2 Supplementary Table

Supplementary Table 1 | Sequences of oligonucleotide primers for real-time RT-PCR analysis in this study.

Gene name	BRAD Number		Primer sequence (5′-3′)
DCD	*Bra018726*	Forward:<br>Reverse:	CAAGGGAGATTGAGGAGCAG<br>TTCGGATCCTTGGTCATCTC
	Bra025184	Forward:<br>Reverse:	CAGCAGCTTGGATACGATCA<br>ACCAAACATCCCAAGAGTGC
LCD	*Bra001131*	Forward:<br>Reverse:	ACCGGACAATTTGGATTTCA<br>GCCGTTTTGCCACCTTAATA
	Bra037682	Forward:<br>Reverse:	CAGCCAATCCCAAGATTCAT<br>TTGTGAGGTCCTGGTTTTCC
	Bra004781	Forward:<br>Reverse:	CCACGGAAAGTGCCATACTT<br>TTCAGGTCTCTTTGCCACCT
	Bra014529	Forward:<br>Reverse:	GCGATTTCAAGTGAGGAAGC<br>TCCCGGATAGACTGGAACAG
	Bra039708	Forward:<br>Reverse:	GGCAAGGCTTCTTGCTCTTA<br>CTTTCCTTGTGGCATCGAAT
	Bra036910	Forward:<br>Reverse:	CCTGCTAACCCAAAGATCCA<br>TATAAAACCAGCGCCAATCC
	Bra009985	Forward:<br>Reverse:	GCGGTGGAACCTACAGAGAG<br>GAGCAAGAAGCTTGGCTGTT
	Bra020605	Forward:<br>Reverse:	GGATTGTTGGTGGGCATATC<br>GTCGGCAGATTCTCTGCTTC
	Bra006115	Forward:<br>Reverse:	GCACTGGTGGAACGGTTAGT<br>GGTTGGAATGACACCAGGAC
	Bra006114	Forward:<br>Reverse:	TGGTCCGGAGATATGGAGAG<br>CCTGGTTCTCCTCCACTGAG
Br_UPB1A	*Bra004465*	Forward:<br>Reverse:	TCAGTTTGGGTGTCGATGAG<br>CCTGAACCATTGTCTGCATAAC
Br_UPB1B	*Bra021395*	Forward:<br>Reverse:	CGCCATTCATAGACGAGTCA<br>TTTCGGTCAAAGCCTGAAAC
Peroxidase	*Bra035235*	Forward:<br>Reverse:	ACTTGCCCTAACGCAGAGAA<br>AGGACACTTCCTCTCCAGCA

	Bra033551	Forward:<br>Reverse:	CTGTCTCATGCGCAGACATT<br>CACTTTGCCTTTCCCAGTGT
	Bra036423	Forward:<br>Reverse:	CGATCGCATTTGCTCAACTA<br>TCTAACGCTTCCGTTTGGTC
	Bra023639	Forward:<br>Reverse:	GTGCACATACCGTTGGTCAG<br>GGTCAACCTGCATCACTCCT
Actin	*Bra028615*	Forward:<br>Reverse:	CTATCCTCCGTCTCGATCTCGC<br>CTTAGCCGTCTCCAGCTCTTGC

3 Supplementary References

Chen, J., Wang, W.-H., Wu, F.-H., You, C.-Y., Liu, T.-W., Dong, X.-J., et al. (2013). Hydrogen sulfide alleviates aluminum toxicity in barley seedlings. *Plant Soil* 362**,** 301-318. doi: 10.1007/s11104-012-1275-7.

Frahry, G., and Schopfer, P. (2001). NADH-stimulated, cyanide-resistant superoxide production in maize coleoptiles analyzed with a tetrazolium-based assay. *Planta* 212**,** 175-183. doi: 10.1007/s004250000376.

Jana, S., and Choudhuri, M.A. (1982). Glycolate metabolism of three submersed aquatic angiosperms during ageing. *Aquat. Bot.* 12**,** 345-354.

Larkin, M.A., Blackshields, G., Brown, N.P., Chenna, R., McGettigan, P.A., McWilliam, H., et al. (2007). Clustal W and Clustal X version 2.0. *Bioinformatics* 23**,** 2947-2948. doi: 10.1093/bioinformatics/btm404.

Letunic, I., Doerks, T., and Bork, P. (2012). SMART 7: recent updates to the protein domain annotation resource. *Nucleic Acids Res.* 40**,** D302-305. doi: 10.1093/nar/gkr931.

Nguyen, H.N., Kim, J.H., Jeong, C.Y., Hong, S.W., and Lee, H. (2013). Inhibition of histone deacetylation alters *Arabidopsis* root growth in response to auxin via PIN1 degradation. *Plant Cell Rep.* 32**,** 1625-1636. doi: 10.1007/s00299-013-1474-6.

Yang, Y., Fan, F., Zhuo, R., Ma, F., Gong, Y., Wan, X., et al. (2012). Expression of the laccase gene from a white rot fungus in *Pichia pastoris* can enhance the resistance of this yeast to H_2O_2-mediated oxidative stress by stimulating the glutathione-based antioxidative system. *Appl. Environ. Microbiol.* 78**,** 5845-5854. doi: 10.1128/aem.00218-12.
